# The Role of Micronutrients in the Infection and Subsequent Response to Hepatitis C Virus

**DOI:** 10.3390/cells8060603

**Published:** 2019-06-17

**Authors:** Sunil Gupta, Scott A. Read, Nicholas A. Shackel, Lionel Hebbard, Jacob George, Golo Ahlenstiel

**Affiliations:** 1 Blacktown Clinical School, Western Sydney University, Blacktown, NSW 2148, Australia; sunil.gupta@live.com.au (S.G.); S.read@westernsydney.edu.au (S.A.R.); 2 Storr Liver Centre, The Westmead Institute for Medical Research, University of Sydney, Westmead 2145, Australia; Jacob.george@sydney.edu.au; 3 Department of Medicine, University of New South Wales, Kensington, NSW 2052, Australia; n.shackel@unsw.edu.au; 4 Department of Molecular and Cell Biology, Centre for Molecular Therapeutics, James Cook University, Australian Institute of Tropical Health and Medicine, Townsville, QLD 4814, Australia; lionel.hebbard@jcu.edu.au; 5 Department of Medicine, Blacktown Hospital, Blacktown, NSW 2148, Australia

**Keywords:** hepatitis C virus, micronutrients, micronutrient deficiency, liver, innate immunity

## Abstract

Micronutrient deficiencies develop for a variety of reasons, whether geographic, socioeconomic, nutritional, or as a result of disease pathologies such as chronic viral infection. As micronutrients are essential for a strong immune response, deficiencies can significantly dampen both the innate and the adaptive arms of antiviral immunity. The innate immune response in particular is crucial to protect against hepatitis C virus (HCV), a hepatotropic virus that maintains chronic infection in up to 80% of individuals if left untreated. While many micronutrients are required for HCV replication, an overlapping group of micronutrients are also necessary to enact a potent immune response. As the liver is responsible for the storage and metabolism of many micronutrients, HCV persistence can influence the micronutrients’ steady state to benefit viral persistence both directly and by weakening the antiviral response. This review will focus on common micronutrients such as zinc, iron, copper, selenium, vitamin A, vitamin B12, vitamin D and vitamin E. We will explore their role in the pathogenesis of HCV infection and in the response to antiviral therapy. While chronic hepatitis C virus infection drives deficiencies in micronutrients such as zinc, selenium, vitamin A and B12, it also stimulates copper and iron excess; these micronutrients influence antioxidant, inflammatory and immune responses to HCV.

## 1. Introduction

Micronutrients are trace elements and vitamins obtained from our diet that are essential to sustain life and optimal physiological function [1,2]. Deficiencies affect over 2 billion people and are largely associated with malnutrition or poor diet [2,3]. Many micronutrients are necessary to elicit an effective immune response to viral infections but are also utilized by viruses such as the hepatitis C virus (HCV) to propagate [4]. HCV is an RNA flavivirus that infects hepatocytes and is typically transmitted through exposure to infected body fluids, including blood transfusion and injecting drug use [5,6]. Like many viruses, HCV has the ability to impede antiviral and apoptotic responses to favor its own persistence [7]. Consequently, approximately 80% of untreated infections progress to chronic hepatitis C (CHC), with persistent viremia and hepatic inflammation [8]. Given that the liver is responsible for the storage and metabolism of many micronutrients such as iron and zinc [9,10], chronic HCV replication has the potential to negatively influence this state. As such, there is a delicate balance between the maintenance of micronutrient stores and micronutrient availability during infection.

HCV infection induces an acute inflammatory response driven by pro-inflammatory cytokines such as IL-6 and TNF-α that can lead to mitochondrial dysfunction and hepatocyte oxidative stress [11]. Chronic hepatocyte damage stimulates persistent inflammation that can lead to the development of liver fibrosis and, ultimately, cirrhosis and cancer [12]. Importantly, numerous micronutrients exist as key components of the hepatic antioxidant response, which can become significantly impaired upon micronutrient deficiency. A prime example is zinc, that is tightly bound to metallothionein (MT) chaperone proteins in the liver [13]. MTs function as intracellular sensors of oxidative stress and heavy metal dysregulation and act to detoxify and scavenge free radicals [14,15].

In CHC, micronutrient deficiency is common, with deficiencies in zinc, vitamin A and vitamin D reported in up to 48.4%, 54.3% and 43% of patients, respectively [16,17,18]. Whilst micronutrient deficiencies may be mediated by HCV replication and associated inflammation, they may be further exacerbated by malnutrition due to lifestyle factors associated with chronic viral hepatitis [19]. Up to 14% of patients with CHC are malnourished, with deficiency of some micronutrients such as zinc reported in up to 90% of patients with compensated cirrhosis and in 98% with decompensated cirrhosis, a manifestation of caloric and protein restriction [20]. This review summarizes the metabolic and immunological roles of the common micronutrients zinc, iron, copper, selenium, vitamin A, vitamin B12, vitamin D and vitamin E. We will comment on the clinical importance of deficiency or excess of these micronutrients, their role in HCV pathogenesis and the associated immune response.

## 2. Zinc

Zinc is an essential trace element important for growth and development. It is found in a variety of foods including meats, cereals, grains, beans and dairy products [21,22]. Up to 10% of the human proteome binds zinc [23]. Protein-bound zinc plays an essential catalytic role in metalloenzyme activity, transcription factor binding and gene regulation [24]. In particular, zinc is required for optimal innate immune defenses including phagocytosis, natural killer cell activity, generation of oxidative bursts, cytokine production and complement activity (reviewed in [25,26]). Zinc distribution within the body is widespread, with the greatest stores found in skeletal muscle (57%), bone (29%) and liver (6%) [9]. Within the blood, 60% of zinc is loosely bound to albumin and 30% of it is tightly bound to macroglobulin [27]. Signs of zinc deficiency include growth retardation, hair loss, diarrhea and delayed development of secondary sexual characteristics [28].

### 2.1. Zinc Deficiency in HCV

In acute HCV infection, inflammatory cytokines such as IL-6 stimulate hepatic zinc uptake via the Zip14 zinc transporter, resulting in transient hypozincemia [29]. Elevated cytosolic zinc up-regulates MT expression, which exerts numerous anti-oxidant and anti-viral effects [13]. In CHC, studies have demonstrated that up to 48.4% of patients develop persistent hypozincemia (<70 µg/dL) [18]. It is thought that, as a result of HCV-mediated mitochondrial dysfunction, the presence of oxidative stress disrupts zinc homeostasis, particularly as it is a signaling molecule and secondary messenger in the reduction–oxidation (redox) process [30]. Importantly, upon viral eradication via either interferon (IFN)-based regimens or direct-acting anti-viral (DAA) therapy, serum zinc levels significantly increase, likely reflecting the resolution of hepatic inflammation and, perhaps, improved gut absorption [31,32].

CHC-related liver fibrosis has also been shown to result in hypoalbuminemia. As 60% of serum zinc is bound to albumin, this results in a decrease in total zinc concentration [18,31]. Furthermore, liver cirrhosis-related portal hypertension may further impair intestinal zinc absorption [33]. This may be compounded by a poor appetite and subsequent malnourishment that often accompanies liver cirrhosis [34].

### 2.2. The Effect of Zinc on HCV Pathogenesis, Immune Response and Treatment

HCV infection stimulates the expression of the antiviral Th1 cytokines IL-2 and IFN-γ which are key drivers of the cytotoxic immune response required to clear acute infection [35]. Human studies show that zinc deficiency leads to a reduction in cytotoxic T cell populations [36], a decrease in natural killer (NK) cell activity [37] and a down-regulation of the Th1 response [38]. In particular, dietary zinc restriction reduces IL-2 and IFN-γ, with no effect on the Th2 cytokines IL-4 and IL-10 [38]. Consequently, low levels of circulating cytotoxic T cells and thymic atrophy due to zinc deficiency may be explained in part, by a reduction in IL-2, a key driver of cytotoxic T cell proliferation and HCV clearance [39]. Importantly, excess zinc supplementation can also reduce the expression of key antiviral cytokines such as IFN-γ by reducing IRF1 expression in regulatory T cells, as observed in vitro [40]. It can lead to impairment of lymphocyte activation and granulocyte chemotaxis and phagocytosis in humans [41]. Consequently, it is vital to measure zinc status prior to zinc supplementation, as excess zinc can interfere with a potent antiviral response.

**Figure 1:**
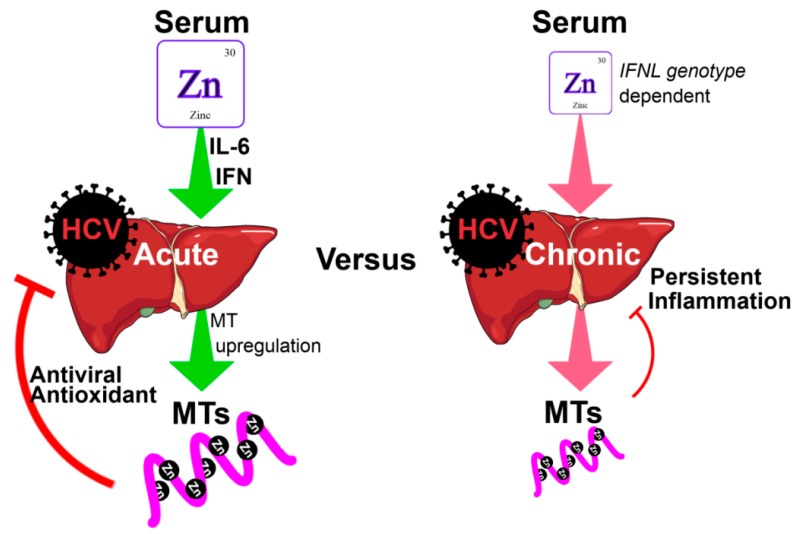
Role of zinc and metallothioneins (MTs) in acute and chronic hepatitis C virus (HCV) infections. In acute HCV infection, pro-inflammatory cytokines such as IL-6 and IFN-λ stimulate the redistribution of serum zinc into the liver. Elevated hepatic zinc stimulates the induction of MTs, which serve as potent antioxidants via their binding and release of zinc, but also display mild antiviral activity. For reasons that remain poorly defined, chronic HCV infection results in low serum and hepatic zinc. Consequently, liver MT expression is reduced, and the liver is subject to chronic inflammation due to persistent viral replication and oxidative stress. Further, chronic hepatitis C (CHC) patients with the *rs12979860* CC interferon lambda (*IFNL*) genotype possess increased serum zinc and demonstrate improved responses to antiviral treatment, supporting the immuno-stimulatory role of zinc.

It has been demonstrated in vitro that zinc may play an important role as a negative regulator of HCV replication in genome-length HCV RNA-replicating cells, albeit via an unknown mechanism [42]. This is possibly via the induction of MTs that possess mild antiviral activity (Figure 1) [13]. Whilst acute HCV infection drives MT induction in vitro, patients with CHC have low serum zinc and low hepatic MT expression [43,44]. This suggests that chronic HCV infection has a markedly different effect on zinc distribution compared to acute HCV infection. Furthermore, low hepatic MT expression is associated with increased hepatic inflammation and fibrosis, suggesting that MTs and zinc protect against chronic inflammation [44]. In support of this, studies have shown that elevated hepatic MT expression is associated with improved liver function as demonstrated by reduced alanine aminotransferase (ALT) and aspartate aminotransferase (AST) levels [45].

Hepatic zinc and MT expression also appear to affect HCV treatment response. In fact, zinc supplementation has been shown to increase hepatic MT expression and enhance the response to IFN-α therapies [46]. Nagamine et al. demonstrated that over a 24-week period of IFN-α therapy, CHC treatment responders had higher pre-treatment serum zinc levels and higher post-treatment hepatic MT expression [47]. Similarly, another study demonstrated that higher baseline zinc levels resulted in a larger decline in serum zinc post-IFN-α treatment, and in these conditions, zinc likely localized to the liver, stimulating MT expression and anti-viral activity [32]. A more recent in vitro study by our group supports these findings, showing that zinc supplementation in MT knockdown cells did not appear to inhibit HCV replication [13].

Liver transcriptomic data and in vitro studies with the Huh-7 hepatoma cell line demonstrated that along with driving MT expression, zinc potently inhibited signaling by IFN-λ3 [43], a cytokine with a central role in the pathogenesis and clearance of HCV infection [43,48]. CHC serum zinc levels and hepatic MT expression exhibited strong inverse correlations with inflammatory and interferon-stimulated genes (ISGs) in the liver [43]. Interestingly, because serum zinc levels were elevated in CHC patients with the IFN-λ3 *rs12979860* CC (responder) genotype, these data suggest that zinc may sensitize the anti-viral response by reducing baseline ISG expression to facilitate strong antiviral responses with minimal interferon refractoriness upon antiviral treatment [49].

## 3. Iron

As the most abundant trace element in the human body, iron plays a key role in DNA and protein synthesis, erythrocyte production, electron transport, cellular respiration, cell proliferation and regulation of gene expression [50]. Dietary iron is absorbed through the divalent metal transporter 1 (DMT1) by duodenal and jejunal enterocytes [51]. It is then exported by ferroportin (FPN) into the bloodstream where it becomes bound to transferrin and is utilized by the muscle and erythroid compartments. In addition, iron is stored as ferritin or hemosiderin in enterocytes, macrophages and hepatocytes [51]. The best-absorbed form of iron (“heme”) is found in meat, poultry and fish, whereas non-heme iron is found in leafy green vegetables, seeds of legumes, fruits and dairy products [52]. Iron deficiency can lead to fatigue, anemia, infertility in females and depression [52]. Being an oxidant with free radical activity, excess iron leads to the breakdown of cellular membranes, ultimately leading to damage in organs such as the liver, kidneys, heart and lungs [53].

### 3.1. Iron Excess in HCV

Iron overload is a prominent feature of CHC, with 10–42% of patients demonstrating hepatic iron accumulation [54,55]. Elevated serum ferritin and transferrin saturation are similarly common in up to 40% of patients [55], where they significantly correlate with hepatic fibrosis [56,57]. Importantly, while serum ferritin can correlate with liver iron, it can also be elevated in the absence of hepatic iron overload and be a marker of liver inflammation [58]. Consequently, because of the inflammatory nature of CHC, liver biopsy is the gold standard for the assessment of hepatic iron overload.

HCV has been shown to influence iron absorption via oxidative stress-mediated down-regulation of hepcidin expression [59,60]. Hepcidin is a peptide hormone produced by the liver that binds FPN to induce its ubiquitination, internalization and degradation [61]. Consequently, HCV-mediated down-regulation of hepcidin results in elevated enterocyte FPN levels and increased intestinal absorption of iron [62]. Fujita et al. have shown that the hepcidin-to-ferritin ratio is significantly lower in HCV patients compared to controls or patients with hepatitis B virus (HBV), suggesting a possible association [63].

### 3.2. The Effect of Iron on HCV Pathogenesis, Immune Response and Treatment

The antiviral role of iron has generated conflicting results in vitro. Using human hepatocyte cell lines, iron has been shown to both enhance [64] and inhibit [65] the replication of HCV. Iron can promote HCV translation through altering the affinity of common cellular factors, the HCV internal ribosome entry site (IRES), via increased expression of eukaryotic initiation factor 3 (EIF3), and the intracellular La ribonucleoprotein [66]. Conversely, iron can inactivate the viral polymerase NS5B [67].

In CHC patients, iron levels have been positively associated with hepatic ALT, suggesting that iron-mediated stimulation of viral replication in vitro may be relevant in vivo [68]. Iron deposition in the liver of HCV patients triggers reactive oxygen species (ROS), which can induce secondary lipid peroxidation and ultimately lead to mitochondrial dysfunction and protein and nuclei acid damage [68,69]. Interestingly, phlebotomy has been shown to reduce liver transaminases and gamma glutamyltransferase (GGT) without affecting HCV viral load [70,71,72].

The antiviral effect of therapeutic phlebotomy in patients with CHC and iron overload has been inconclusive. Fargion et al. studied 114 patients with hepatic iron concentrations (HIC) ≥700 mcg/g in men and ≥500 mcg/g in women [73]. Compared with IFN-α therapy alone, those who received phlebotomy followed by IFN-α showed a trend towards a greater sustained virologic response (SVR) at an odds ratio of 2.32 (*p* = 0.082) [73]. In agreement with these data, lower HIC and serum ferritin were measured in patients who responded to IFN-α treatment [74]. Conversely, baseline HIC had no effect on the response to IFN-α therapy in beta-thalassemia major patients with CHC and transfusion-acquired iron overload [75]. With regards to previously treated CHC non-responders to IFN-α therapy, phlebotomy had no effect on SVR following re-treatment; however, there was a decrease in liver injury with reduced AST and improved necro-inflammatory changes on liver biopsy [76].

## 4. Selenium

In trace amounts, selenium is indispensable for the maintenance of good health [77]; it can be obtained from cereals, grains, fish, meat and dairy products [21,22]. Selenium is a constituent of glutathione peroxidase (GPx), an enzyme that protects against damage induced by free radicals at a cellular level [78]. Selenium is also essential in augmenting antiviral immunity [79] and assisting in the detoxification of liver enzymes [80]. Other major selenium-containing proteins in blood include selenoprotein-P and albumin [81,82,83]. Deficiency can lead to fatal cardiomyopathies such as Keshan disease [84] and degenerative disorders such as Kashin–Beck disease [85].

### 4.1. Selenium Deficiency in HCV

The presence of selenium deficiency in CHC remains uncertain. CHC infection has been shown to result in a reduction of blood selenium levels (99.8 ± 11.0 μg/L in CHC versus 117.5 ± 15.7 μg/L in healthy controls) that decrease even further following the development of HCV-related cirrhosis (84.7 ± 16.4 μg/L) [86]. These data are supported by studies that demonstrate a decline in serum selenium levels in proportion to the degree of hepatic fibrosis, as determined by tissue sampling [87]. In addition, GPx activity is reduced along with selenium levels in CHC (*r* = 0.374, *p* = 0.148), providing a possible mechanism by which CHC stimulates oxidative stress due to selenium deficiency [87]. While others have found no significant reduction in serum selenium in CHC patients [88], alcohol intake was a major confounding variable as it affects selenium levels [89].

With regard to the mechanism by which selenium becomes deficient in CHC patients, Thuluvath et al. have demonstrated that urinary excretion of selenium was not significantly different in cirrhotic patients compared to controls, although this study was not specific to viral hepatitis [90]. Nonetheless, these data suggest that reduced selenium levels may be secondary to malabsorption in the small intestine [87]. Further studies are required in CHC patients to determine the underlying cause for selenium deficiency.

### 4.2. The Effect of Selenium on HCV Pathogenesis, Immune Response and Treatment

RNA viruses, including HCV, encode selenium-dependent *GPx* genes [91,92]. In view of this finding, it is possible that the sequestration of selenium to facilitate viral replication could generate a shortage of circulating selenium [93]. In vitro, HCV can inhibit the expression of gastrointestinal-GPx, a GPx that is also expressed in the liver, resulting in an increase in viral replication [94]. These data are supported by clinical data demonstrating a negative correlation between HCV viral load and selenium (*r* = −0.730) and GPx activity (*r* = −0.675) [95]. In that study, plasma GPx activity, plasma selenium and erythrocyte selenium levels were significantly lower in CHC patients than in controls; however, hepatic selenium was not measured [95].

In CHC patients, low serum selenium levels were found to positively associate with low GPx activity (*r* = 0.374, *p* = 0.0148) but not with HCV genotype or HCV-RNA load [87]. While these data do not indicate that HCV directly decreases selenium, they raise the possibility of selenium replacement as a therapeutic supplement to boost anti-oxidant and antiviral defenses. This supports animal models of HCV infection where reduced selenium levels resulted in the accumulation of lipid peroxides [96], which led to increased expression of VEGF and IL-8, accelerating the development of HCC [96], whilst also stimulating hepatic fibrosis via hepatic stellate cell activation [97].

The effect of selenium has not been examined in the context of HCV treatment but has been assessed for its anti-inflammatory properties. Berkson et al. analyzed the effect of selenium alpha-lipoic acid and silymarin supplementation in three CHC patients and demonstrated an improvement in ALT [93]. A more recent randomized, placebo-controlled, double-blinded trial assessed the effect of ascorbic acid (500 mg), vitamin E (945 IU) and selenium (200 µg) versus placebo for six months. During supplementation, the group administered the antioxidants had higher erythrocyte GPx; however, no differences were observed in serum ALT or HCV viral load [98]. While limited, these data suggest that selenium supplementation alone may not be adequate to suppress inflammation in CHC.

## 5. Copper

Copper is an essential trace element that is present in mineral-rich foods like meats, oysters, nuts, seeds, dark chocolate and whole grains [52]. The main protein carrier of copper in the human body is ceruloplasmin (95%), whilst a small amount of copper is bound to albumin [99,100]. Once absorbed from the digestive tract, copper is transported to the liver and is essential for erythrocyte production [101]. Up to 50% of copper content is stored in muscles and bone, 8% in the brain and 8–15% in the liver [100]. Copper has the ability to accept electrons, which makes it important in redox processes [102]. As such, it is a functional component of several essential enzymes including cytochrome oxidase and superoxide dismutase, which serve to catalyse the reduction of oxygen molecules to water and of free radicals to hydrogen peroxide [102]. Copper deficiency can cause anemia, defective keratinization and pigmentation [103], whilst copper excess can cause acne, alopecia, strokes, pancreatic dysfunction and osteoporosis [104].

### 5.1. Copper Excess in HCV

Acute HCV infection stimulates an increase in serum copper [105], which is further exacerbated in CHC and fibrotic liver disease [105,106,107]. Hepatic copper is increased in CHC [107], where it is primarily bound to MTs (Cu–MTs), thus contributing to hepatic copper overload [108]. The mechanism by which HCV stimulates copper accumulation in the liver remains unknown, however, Cu–MTs have been shown to stimulate hydroxyl radical generation in rats, thus driving liver damage and, perhaps, fibrosis [109,110]. In a recent human study, hepatic copper content increased significantly as hepatic fibrosis advanced and correlated positively with bilirubin (*r* = 0.466, *p* = 0.0023) and type IV collagen (*r* = 0.402, *p* = 0.0086) [109]. As copper is excreted solely in the bile, HCV-mediated inhibition of bile acid secretion may indeed result in the retention of biliary copper [111,112]. Interestingly, copper and zinc metabolism are strongly linked in the liver. Zinc over-supplementation can lead to copper deficiency via MT-mediated inhibition of copper absorption in the gut [101,113].

### 5.2. The Effect of Copper on HCV Pathogenesis, Immune Response and Treatment

While there are no direct links between copper excess and the pathogenesis in CHC, unbound copper efficiently catalyzes the formation of ROS and is a potential driver of oxidative liver damage [114]. Oxidative stress is further aggravated by a decline in glutathione in CHC patients, which serves as a key hepatic antioxidant by binding free copper [115].

No clinical studies have examined the antiviral role of copper supplementation or depletion in humans; however, copper in various forms can exhibit antiviral properties [116,117]. In particular, cuprous oxide nanoparticles (CO-NPs), a p-type semiconductor that possesses unique physical and chemical properties [118,119], have been shown to inhibit the infectivity of HCV cell cultures at a non-cytotoxic concentration. In addition, CO-NPs can inhibit the attachment and entry of genotype 1a, 1b and 2a HCV pseudoparticles (HCVpp), with no effect on HCV replication [120].

## 6. Vitamin A

Vitamin A represents a group of fat-soluble retinoids including retinol and retinoic acid. The majority of liver vitamin A is stored in hepatic stellate cells (HSCs) and hepatocytes [10]. Retinoic acid (RA) is thought to play a role in potentiating the innate immune response by binding to cellular retinoic acid-binding protein (CRABP) 1 and 2 [121]. Vitamin A can be obtained from vegetables, dairy products, eggs and fruits [21,22].

### 6.1. Vitamin A Deficiency in HCV

In the setting of CHC, vitamin A deficiency is common. A study of 199 treatment-naive CHC patients found that serum vitamin A was significantly lower compared to healthy controls (256 ng/mL versus 742 ng/mL; *P* < 0.0001) [122]. Peres et al. showed that in patients with CHC, vitamin A deficiency was present in 54.3% of patients, with a serum retinol decline in those with cirrhosis and hepatocellular carcinoma (HCC) [16]. In support, a 2002 study by Yadav et al. found diminished liver retinol levels in CHC patients with moderate to severe fibrosis, compared with those with mild fibrosis [123]. Notably, because only free retinol was assessed, any inference regarding total liver vitamin A stores cannot be made. Nonetheless, these data suggest that vitamin A deficiency is associated primarily with the development of liver fibrosis. HSCs lose their retinoid content once they become activated as a consequence of CHC, resulting in the formation of a collagenous extracellular matrix that drives liver fibrosis [10]. Consequently, they are a likely candidate for the drastic reduction of hepatic retinol observed in fibrotic CHC patients.

### 6.2. The Effect of Vitamin A on HCV Pathogenesis, Immune Response and Treatment

Vitamin A has been shown to both promote and inhibit HCV replication in vitro. Retinoic acid binding to CRABP1 can activate lipid metabolism gene expression in hepatocytes, thus providing a platform for HCV replication complexes and subsequent viral propagation [124,125]. A separate study has demonstrated that administration of all-trans-retinoic acid (ATRA), an active metabolite of vitamin A, results in the down-regulation of the HCV replicon, at least in part via the induction of GPx [94]. In addition, ATRA has been shown to increase the expression of IFN-α receptor IFNAR1, enhancing the antiviral effect of IFN-α.

Because of its antiviral effect in vitro, a recent study administered ATRA in combination with IFN-α to a cohort of CHC patients that had previously failed therapy [126]. While none of the patients achieved SVR, ATRA demonstrated a direct antiviral and synergistic effect with pegylated-IFN-α, resulting in a reduction in HCV viral load 12 weeks after the completion of treatment [127]. A possible mechanism by which ATRA stimulates the antiviral response is through the up-regulation of the dsRNA helicase enzyme retinoic acid-inducible gene-I (RIG-I) [128]. RIG-I is responsible for HCV dsRNA recognition and subsequent IFN-α and IFN-λ production as part of the innate antiviral response in the liver [126,129]. In agreement, combined vitamin A and D deficiency was found to be a strong independent predictor of nonresponse to antiviral therapy [122]. It should be noted that vitamin A supplementation in clinical practice is limited by its possible hepatotoxicity [130].

## 7. Vitamin B12

Vitamin B12 (cobalamin) is a water-soluble vitamin obtained through the consumption of meat, liver, fish, dairy products and fortified cereals [131,132]. It is co-absorbed with intrinsic factor, a product of gastric parietal cells, in the terminal ileum. Cobalamin is vital for normal neurologic function, erythrocyte production and DNA synthesis [131,132]. Deficiency can result in hyperpigmentation, vitiligo, glossitis, anemia, cognitive impairment, gait abnormalities and areflexia [133]. Importantly, vitamin B12 is stored in high concentration in the human liver [134]. A review of the literature did not reveal any studies with data to suggest the presence of vitamin B12 deficiency in CHC.

### The Effect of Vitamin B12 on HCV Pathogenesis, Immune Response and Treatment

HCV utilizes a cap-independent initiation of translation through an IRES that is present in the viral 5’ untranslated region (UTR) [135]. As vitamin B12 is highly concentrated in the liver [134] and has a strong affinity for RNA pseudoknots [136], the effect of cobalamin has been examined with respect to IRES function. Vitamin B12 was shown to inhibit HCV IRES-dependent initiation of translation in vitro and was suggested to represent an evolutionary mechanism serving to limit HCV replication and promote persistence [137]. However, it also suggests that vitamin B12 may offer a therapeutic option for CHC treatment [137].

Human studies have demonstrated conflicting results regarding baseline serum vitamin B12 and response to IFN-based therapy. While Rosenberg et al. found significantly lower B12 in non-responders (NR) when compared to responders (R) (262 pM NR versus 331 pM R) [138], Mechie et al. showed the opposite effect (616 ng/L NR versus 333 ng/L R) [139]. Elevated B12 measured in cirrhotic patients may, however, reflect their poor hepatic vitamin B12 clearance [140]. Consequently, cirrhosis may impair responses to IFN-based therapies and increase serum vitamin B12, independently. Supporting the beneficial role of baseline vitamin B12, serum homocysteine levels are reduced (<16 µmol/L) in responders to pegylated IFN plus ribavirin therapy [141]. Homocysteine accumulates with vitamin B12 deficiency, and is considered a sensitive indicator of vitamin B12 levels [142].

To assess the role of vitamin B12 in treatment response, studies have examined supplementation in combination with standard pegylated-IFN-α plus ribavirin therapy. Higher rates of SVR were observed 24 weeks after therapy completion in patients receiving vitamin B12 supplementation [143,144], supporting an association between pre-treatment vitamin B12 levels and SVR [138].

## 8. Vitamin D

Vitamin D is a secosteroid hormone that plays a key role in calcium and bone homeostasis [145]. Vitamin D_3_ (VD_3_) is synthesized in the skin from 7-dehydrocholesterol, a process which depends on sunlight [146]. VD_3_ is then converted in the liver to 25-dihydroxyvitamin D_3_ (25(OH)VD_3_) which is metabolized in the kidneys to 1,25(OH)_2_VD_3_, the most physiologically active VD_3_ metabolite [145,146,147,148]. Recently, evidence has emerged that vitamin D has anti-fibrotic, anti-inflammatory and immunomodulatory properties. More specifically, it inhibits T cell proliferation, expression of interleukin-2, expression of IFN-γ and CD8 T-lymphocyte-mediated cytotoxicity [146]. These extra-skeletal effects are relevant in the pathogenesis and treatment of many causes of chronic liver disease [147,149].

### 8.1. Vitamin D Deficiency in HCV

Vitamin D deficiency (<20 ng/mL) and suboptimal levels (20–30 ng/mL) are widespread across CHC populations, far exceeding the worldwide rate of insufficiency of 33% [150]. The presence of vitamin D deficiency nonetheless varies among CHC populations, with values below 30 ng/mL measured in 77% (Spain, [149]), 66.2% (Brazil, [151]), 56.9%, (USA, Caucasian [152]) and 85.8% (USA, African-American [152]) of infected persons. Importantly, oral supplementation can significantly improve 25(OH)VD_3_ deficiencies upon treatment [149]. Studies have assessed vitamin D deficiency in CHC, finding that low Vitamin D levels were linked to severe fibrosis [153]. Mechanistically, vitamin D deficiency has been suggested to predispose CHC patients to higher levels of oxidative stress and nitric oxide metabolites [154].

### 8.2. The effect of Vitamin D on HCV Pathogenesis, Immune Response and Treatment

The direct antiviral role of Vitamin D_3_ and its metabolites remains uncertain due to conflicting in vitro studies. While vitamin D_3_ and its metabolites calcifediol and calcitriol have been shown to inhibit viral replication by up to 80% [155,156], other studies have found no direct antiviral effect [157]. Nonetheless, all in vitro studies support the role of vitamin D_3_ in strengthening the IFN response by increasing STAT1 nuclear localization, DNA binding and ISG expression [155,156,157]. Interestingly, HCV infection markedly increased calcitriol levels in cell culture due to the reduction of 24-hydroxylase, the enzyme responsible for the first step of calcitriol breakdown [155]. Whether this is a virus-specific mechanism or a component of the hepatic antiviral response is not yet fully understood.

The impact of pre-treatment vitamin D deficiency on SVR following IFN therapies has yielded mixed results. It was found to be a predictor of response in genotype 1 (HCV-1) and HCV-2/3 patients from Europe [153] and Asia [158] and HCV-4 patients from Africa [159]. In particular, vitamin D levels greater than 18 ng/mL were associated with a higher SVR rate (65%) when compared with lower levels (38.5%) [160]. However, other studies conducted mostly in HCV-1 patients found no clear association [149,161,162].

Studies examining vitamin D supplementation with IFN-α plus ribavirin therapy have shown improved rates of SVR (24 weeks after completion) in genotypes 1, 2 and 3 [158,163]. However, a study in India of patients with genotypes 1 and 4 that were administered vitamin D supplementation alongside pegylated-IFNα plus ribavirin therapy found no association [164]. Cohort heterogeneity (patient inclusion criteria) and characteristics of vitamin D assessment (laboratory methods and cut-off values) likely contribute to differences in the findings of different studies.

To date, only one study has evaluated vitamin D levels in patients treated with DAAs, demonstrating that vitamin D levels increased mildly but not significantly following DAA treatment [17]. Furthermore, neither the pre-treatment level nor the change in levels during DAA therapy was associated with SVR [17]. Although vitamin D may be relevant in the treatment of chronic hepatitis C, further randomized, placebo-armed studies are required in order to confirm whether vitamin D supplementation improves the SVR, particularly in the setting of DAA therapy.

## 9. Vitamin E

Vitamin E is a natural lipid-soluble antioxidant thought to support endogenous defenses against oxidative stress resulting from increased formation of ROS [166]. Vitamin E can be obtained from the diet through oils, fats, vegetables, fish and fruit [21,22]. While studies show diminished vitamin E levels in viral hepatitis, these were not limited to HCV infection [166,167].

### The Effect of Vitamin E on HCV Pathogenesis, Immune Response and Treatment

The anti-oxidant effect of vitamin E on HCV treatment has been trialed in various studies. High-dose vitamin E treatment (twice the daily dose of 400 IU) in patients with CHC refractory to IFN-α therapy showed significant reductions in AST and ALT by eight weeks. This was temporary, as transaminases increased after treatment cessation [165]. In 2014, a study administering 400 IU of vitamin E twice daily to a group of patients with genotype 3 CHC similarly showed a reduction in serum ALT after 12 weeks, from 122.6 ± 80.1 IU/L to 68.4 ± 25.3 IU/L, *p* = 0.016 [168]. These data are supported by viral hepatitis studies demonstrating a significant reduction in vitamin E in patients with elevated hepatic inflammation (ALT) [166].

Falasca et al. demonstrated that a silybin–phospholipid and vitamin E complex (SPV complex) in CHC resulted in a persistent reduction in serum ALT (*p* = 0.02) and AST (*p* = 0.01). There were also a significant increase in IL-2 (*p* = 0.03) and a decrease in IL-6 (*p* = 0.02), suggesting that the complex exerted hepato-protective and anti-inflammatory effects [169]. In 2003, a group analyzed the role of vitamin E in oxidative stress in CHC patients who had partially responded to previous treatment. The level of thioredoxin (TRX), a stress inducible multifunctional protein that is secreted during oxidative stress, was compared pre- and post-treatment with vitamin E 500 mg for three months. The results revealed a marked improvement, with reduction in TRX and ALT [170].

## 10. Conclusions

Micronutrients play a significant role in HCV infection and replication, associated inflammation and fibrosis, and response to therapy (Table 1). Micronutrient deficiencies are common in CHC, with deficiencies in zinc, vitamin A and vitamin D reaching rates close to 50% [16,17,18]. In patients with CHC-related liver disease, deficiency of some micronutrients such as zinc can reach 90% in compensated cirrhosis [20]. This remains relevant in the epidemiology of hepatitis C infections, with developing countries accounting for over 95% of the worlds’ malnourished population [171], whilst having some of the highest rates of CHC [172]. Furthermore, as demonstrated in our review, the virus itself may exacerbate some of these deficiencies.

Zinc supplementation is perhaps most beneficial, as zinc has been shown to negatively regulate HCV replication and reduce copper excess via activation of MTs in the gut; it also shows promise in the setting of DAA therapy. Selenium and vitamin E supplementation have not yielded promising results, with minimal and often temporary improvements in liver transaminases and no effect on HCV viral load. On the other hand, vitamin B12 replacement appears promising, showing a strong correlation with SVR, although it is yet to be studied in the setting of DAA therapy. Deficiencies in vitamin A and D are independent predictors of nonresponse to antiviral therapy; however, further research is required regarding their use as supplements.

Phlebotomy for iron overload in the setting of CHC does not appear to have therapeutic benefit in achieving SVR, whilst there are no studies assessing chelation therapy for copper overload in CHC. Copper nanotechnology is promising for the production of anti-viral agents; however, no human studies to date have been conducted.

With micronutrient deficiencies common in the setting of CHC, supplementation with zinc or vitamin B12 alongside conventional therapies should be considered, particularly in developing nations with high rates of malnutrition and limited access to resources [172]. Despite the high cure rate with direct-acting antivirals for HCV, the high incidence of micronutrient deficiencies supports the notion of screening and supplementation, as the impact of these micronutrients reaches far beyond HCV alone.

## Figures and Tables

**Table 1:** Summary of the role of micronutrients in HCV infection and the immune response.

Micronutrient	Mechanism of Deficiency/Excess	Role in HCV Life Cycle	Role in Tissue Damage / Fibrosis	Role in Treatment Response
**Zinc** **(Deficiency)**	Acute HCV stimulates IL-6 induction, stimulating hepatocyte uptake of zinc via the Zip14 zinc transporter [29].	Zinc is a negative regulator of HCV replication in genome-length RNA-replicating cells [13,42].	Zinc inhibits proliferation and collagen synthesis in HSCs by increasing matrix metalloproteinase 13 [171]. Promotes apoptosis of HSCs and reduces type-IV collagen [172]. Inhibits IFN-λ3 [43].	Zinc supplementation reduces HCV replication in vitro, improves response to IFN-α [42,45,46] and results in higher rate of HCV clearance [173,174].
**Iron** **(Excess)**	Through HCV protein-mediated oxidative stress, hepcidin is lowered, increasing FPN-mediated iron absorption [175].	Iron promotes HCV via initiation factor 3, La proteins, and binding of cellular factors to HCV IRES [66]. Inhibits NS5B polymerase activity [67].	Iron deposition in the liver of HCV patients triggers reactive oxygen species, inducing lipid peroxidation and mitochondrial dysfunction [68,69]. Iron is associated with a higher prevalence of HCC in HCV patients [69].	Phlebotomy modestly increases SVR [73]. In IFN therapy non-responders, reduces AST and necro-inflammation [76].
**Selenium** **(Deficiency)**	Possible malabsorption [87,90] or viral sequestration [93].	HCV inhibits expression of selenium-dependent GPx, promoting intracellular HCV propagation and higher viral loads [94,95].	Selenium levels decline in proportion with hepatic fibrosis [87] and result in accumulation of lipid peroxides. This leads to expression of VEGF and IL-8, accelerating the growth of HCC [96].	Selenium with alpha-lipoic acid and silymarin improves ALT [93]. When given with vitamin E and ascorbic acid, it does not affect ALT or HCV RNA viral load [98].
**Copper** **(Excess)**	Hepatic copper-MT accumulation contribute to copper overload [108]. Reduction in biliary copper secretion [112]	Cuprous oxide nanoparticles (CO-NPs) inhibit the infectivity of HCV in vitro [120].	Hepatic copper increases with hepatic fibrosis and correlates positively with type IV collagen [108]. MT-bound copper stimulates hydroxyl radicals in rats, driving liver damage and fibrosis [109].	No data available.
**Vitamin A** **(Deficiency)**	No clear mechanism identified.	HCV replication is up-regulated in cells expressing CRABP1 via lipid droplet formation [125]. ATRA reduces viral replication [94].	Diminished liver retinol levels are found in CHC patients with moderate to severe fibrosis compared to those with mild fibrosis [123]	ATRA mediates retinoic acid-inducible gene-I [128], leading to transcription of type-1 IFNs, enhancing the effect of IFN-α on HCV [126,129].
**Vitamin B12** **(Deficiency)**	No clear mechanism identified.	HCV may use a virally encoded protein or cellular factor, such as B12, that targets HCV IRES to regulate translation [137].	No clear association identified.	Vitamin B12 supplementation with pegylated-IFN-α and ribavirin therapy improves SVR [143,144].
**Vitamin D** **(Deficiency)**	No clear mechanism identified.	Vitamin D3 inhibits HCV replication via expression of IFN-β [155]. No direct antiviral effect [157].	Low vitamin D levels in CHC are linked to severe fibrosis [153] and higher levels of nitric oxide metabolites [154].	Vitamin D predicts response in HCV-2/3 patients from Europe [153] and Asia [158], and in HCV-4 patients from Africa [159]. Studies in HCV-1 found no association [149,161,162].
**Vitamin E** **(Deficiency)**	No clear mechanism identified.	No clear association identified.	No clear association identified.	High-dose vitamin E results in reductions in ALT and AST [165,168]. In combination with silybin–phospholipid, vitamin E reduced IFN-γ, TNF-α and IL-6, suggesting an anti-inflammatory effect [169].

ALT, alanine aminotransferase; AST, aspartate aminotransferase; ATRA, all-trans-retinoic acid; CRABP1, cellular retinoic acid-binding protein; FPN, ferroportin; GPx, glutathione peroxidase; HSC, hepatic stellate cell; HCC, hepatocellular carcinoma; IFN, interferon; IRES, internal ribosome entry site; MAPK, mitogen-activated protein kinase; SVR, sustained virologic response.
